# Estradiol, Estrone and Ethinyl Estradiol Metabolism Studied by High Resolution LC-MS/MS Using Stable Isotope Labeling and Trapping of Reactive Metabolites

**DOI:** 10.3390/metabo12100931

**Published:** 2022-09-30

**Authors:** Kahina Chabi, Lekha Sleno

**Affiliations:** Chemistry Department, Université du Québec à Montréal, P.O Box Downtown Station, Montreal, QC H3C 3P8, Canada

**Keywords:** metabolism, reactive metabolites, LC-MS/MS, estradiol, estrone, ethinyl estradiol, stable isotope labelling, structural elucidation

## Abstract

Biotransformation reactions that xenobiotics undergo during their metabolism are crucial for their proper excretion from the body, but can also be a source of toxicity, especially in the case of reactive metabolite formation. Unstable, reactive metabolites are capable of covalent binding to proteins, and have often been linked to liver damage and other undesired side effects. A common technique to assess the formation of reactive metabolites employs trapping them in vitro with glutathione and characterizing the resulting adducts by liquid chromatography coupled to tandem mass spectrometry (LC-MS/MS). Some endogenous compounds, however, can interfere with xenobiotic metabolites of interest, making the analysis more difficult. This study demonstrates the usefulness of isotope-labeled compounds to detect and elucidate the structures of both stable metabolites and trapped adducts of three estrogen analogs using an untargeted LC-MS/MS workflow. The metabolism of estradiol, estrone and ethinyl estradiol was investigated. Unlabeled and deuterated versions of these three compounds were incubated with human or rat liver microsomes in the presence of two different trapping agents, namely glutathione and *N*-acetylcysteine. The detection of closely eluting deuterated peaks allowed us to confirm the formation of several known metabolites, as well as many previously uncharacterized ones. The structure of each adduct was elucidated by the detailed analysis of high-resolution MS/MS spectra for elucidating fragmentation pathways with accurate mass measurements. The use of isotopic labeling was crucial in helping confirm many metabolites and adduct structures, as well as removing endogenous interferences.

## 1. Introduction

Estrogens are involved in many key biological processes in development, growth and sexual maturation in humans and animals. The two main estrogens found in humans are 17β-estradiol (E2) and estrone (E1) [1]. In 1943, the FDA (U.S. Food and Drug Administration) authorized the use of a synthetic hormone 17α-ethinyl estradiol (EE2), a structural analog of E2, for specific therapies [2]; moreover, it has been the main active ingredient used in hormonal contraceptives since 1960 [3]. Although endogenous and synthetic estrogens have been shown to have benefits, including reduced risk of osteoporosis and coronary heart disease [4], they have also been linked to potential toxicity for humans and the environment. Numerous studies have shown that estrogens cause the development of many cancers in humans and animals (breast, endometrial, liver and kidney cancer) [5,6]. Excreted estrogens in the environment are also a major concern, as they harm the development and behavior of many aquatic species [7]. Therefore, it is important to investigate the deleterious effects of estrogens and other related xenobiotics to which we may be exposed.

In vivo and in vitro studies have shown that these different hormones undergo significant oxidative metabolism in humans and rats [8]. This phenomenon is induced by cytochromes P450 (CYP) enzymes, dependent on nicotinamide adenine dinucleotide phosphate (NADPH) [9,10]. Endogenous and exogenous estrogens are known to metabolize into catechols, which can easily be oxidized to form reactive ortho-quinones [11]. Zhu et al. have developed a GC-MS method to identify several reactive metabolites formed via NADPH-dependent oxidative metabolism of E1 and E2, in the presence of microsomal fractions of the human liver and placenta [11]. Many metabolites were detected, such as 4-OH-E2, 15α-OH-E2, 16α-OH-E1, 16β, 17α-OH-E2 and 2-methoxy-E2, including in urine samples from pregnant and non-pregnant women, in addition to other well-known metabolites 2-OH-E1/E2, 2-methoxy-E1 and 16α-OH-E2 [11]. Although there is little information on the oxidative metabolism of EE2, it has been reported that this hormone is highly oxidized by human cytochromes P450 forming mainly 2-OH-EE2 [12]. The 2-hydroxy derivatives can then be methylated [12]. If not properly eliminated, these polar metabolites could induce toxicity. Indeed, estrogen metabolites have been linked to increasing concentrations of reactive oxygen species (ROS) which, by binding to DNA, alter immune functions leading to autoimmune diseases [13]. In hamsters, mice and rats, it has been shown that catechols formed from E1 and E2 are responsible for DNA adduct formation by binding to adenine or guanine [14]. The 16α-OH-E1 has been linked to multiple diseases and is able to bind to proteins causing severe tissue damage. Via a “bottom-up” proteomics approach using LC-ESI-HRMS/MS of tryptic peptides, numerous adduction sites of 16α-OH-E1 to albumin have been identified [15].

Mass spectrometry, coupled to either liquid or gas chromatography, is the most common method used in the identification of endogenous and exogenous metabolites in complex biological samples [16]. LC-MS with electrospray ionization (LC-ESI-MS) is the method of choice for metabolite analysis, with the advantage over GC-MS in that sample preparation and analysis is usually simpler and more universally applicable to biological samples. The development of these techniques has provided new tools for the study of sex steroids, providing new information on the synthesis of steroid hormones and their metabolic pathways [16]. As reactive metabolites are highly unstable, it is useful to trap them with small nucleophilic reagents forming stable adducts for analysis [17]. Glutathione (GSH) is a common endogenous nucleophile used as a trapping reagent to study the formation of reactive metabolites. GSH acts as a detoxifying antioxidant in vivo by covalently binding to reactive metabolites, neutralizing their deleterious effects, and leading to their elimination [17,18]. Other similar scavenging reagents have also been used, such as N-acetylcysteine (NAC) and cysteine (Cys) [18]. Butterworth et al. have identified in vitro glutathione conjugates involved in the nephrotoxicity of estradiol, namely 2-hydroxy1-glutathione-S-yl-17β-estradiol, 2-hydroxy-4-glutathione-S-yl-17β-estradiol and 2-hydroxy-1,4-bis-glutathione-S-yl-17β-estradiol [19]. In metabolism studies, the presence of interferences from the biological matrix can make the identification of reactive metabolites complex, making it difficult to accurately identify metabolites in a large, complex set of data. Therefore, using isotope labeling is a good way to address these different problems. Chokkathukalamal et al. have expertly reviewed the difficulties encountered during the study of reactive metabolites and the importance of isotopic labeling to validate and characterize new metabolites by facilitating the interpretation of MS/MS fragmentation spectra [20].

In this study, the oxidative metabolism of E1, E2 and EE2 was evaluated using human and rat liver microsomal incubations, using isotope labeling for the confirmation of formed metabolites and supporting the elucidation of their structures. Liquid chromatography coupled with high-resolution tandem mass spectrometry (LC-HRMS/MS) was performed to characterize all detected adducts formed, using accurate mass measurements, isotope labeling and collision-induced dissociation (CID) fragmentation data. In addition, two trapping agents were used to study the formation of reactive metabolites: GSH and N-acetyl cysteine (NAC). NAC adducts of these compounds have not been investigated previously, and the comparison of results from GSH and NAC helps confirm formed adducts. The use of deuterated analogs helps to distinguish real metabolites from the background biological matrix which has numerous potential interferences, especially for hormone or steroid molecules, as well as helping in the structural elucidation of the detected metabolites and adducts of interest.

## 2. Materials and Methods

### 2.1. Materials

Three estrogen analogs: 17β-Estradiol [E2, 3,17β-Dihydroxy-1,3,5 (10)-estratriene], Estrone [E1, 1,3,5 (10)-Estratrien-3-ol-17-one] and 17α-Ethinyl estradiol [EE2, 17α-Ethinyl-1,3,5 (10)-estratriene-3,17β-diol] were purchased from Sigma-Aldrich (Oakville, ON, Canada). Their deuterated (d4) versions: 17β-Estradiol-d4 [E2-d4, 3,17β-Dihydroxy-1,3,5 (10)-estratriene-2,4,16,16-d4], Estrone-d4 [E1-d4, 1,3,5 (10)-Estratrien-3-ol-17-one-2,4,16,16-d4] and 17α-Ethinyl estradiol-d4 [EE2-d4, 17α-Ethinyl-1,3,5 (10)-estratriene-3,17β-diol-2,4,16,16-d4] were purchased from CDN Isotopes (Pointe-Claire, QC, Canada). Human liver microsomes (HLM) were purchased from Bioreclamation IVT (Westbury, NY, USA). Rat liver microsomes (RLM) were purchased from Corning (Corning, NY, USA). Glutathione (GSH) and N-acetyl-L-cysteine (NAC) were purchased from Sigma-Aldrich (Oakville, ON, Canada), as well as HPLC-grade acetonitrile (ACN), methanol (MeOH), LC-MS-grade formic acid. Ultrapure water was from a Millipore Synergy UV system (Billerica, MA, USA).

### 2.2. In Vitro Incubations

The three estrogens analogs were incubated with their deuterated (d4) versions in a ratio of 1:1 as well as separately at 10 μM, in the presence of human and rat liver microsomes with either GSH or NAC (2.5 mM), in a phosphate buffer at pH 7.4 (100 mM) containing NADPH-regenerating system (5 mM MgCl_2_, 0,5 mM NADP+, 10 mM glucose-6-phosphate and 2 units/mL glucose-6-phosphate dehydrogenase). Both GSH and NAC were used in the human incubations, whereas GSH trapping was also studied in the rat samples. After 1 h at 37 °C, adding an equal volume of cold acetonitrile quenched the reaction; samples were then centrifugated for 8 min at 14,000 rpm, at 4 °C. The supernatants were dried under vacuum and reconstituted with 5% acetonitrile prior to LC-MS/MS analysis. Control samples were prepared without trapping agent or one of the analogs, as well as incubations quenched before adding the NADPH regeneration system. The NADPH regeneration system is added to incubations to continuously produce NADPH during the incubation time as a cofactor for oxidative metabolism.

### 2.3. LC-HRMS/MS Analysis and Data Processing

Samples were analyzed on a Nexera UHPLC (Shimadzu, Columbia, MD, USA) coupled to a quadrupole time-of-flight (TripleTOF^®^ 5600) mass spectrometer (Sciex, Concord, ON, Canada). Water and acetonitrile, containing 0.1% formic acid, were used for gradient elution on an Aeris PEPTIDE XB-C18 column (100 × 2.1 mm, 1.7 μm) preceded by a SecurityGuard™ ULTRA C18 cartridge (2.1 × 2 mm) (Phenomenex^®^, Torrance, CA, USA), at 0.30 mL/min with a column temperature of 40 °C, and injection volume of 20 μL. The HPLC gradient started at 5% B and was held for 1 min, increased linearly to 85% for 18 min and to 95% for 0.90 min.

TOF-MS acquisition was performed with 300 ms accumulation time, followed by collision-induced dissociation (CID) MS/MS (in high sensitivity mode) using data-dependent acquisition (top five ions, with dynamic background subtraction) (150 ms each), used in positive electrospray mode to acquire high-resolution MS/MS. Nitrogen was used as collision gas and the collision energy was 30 ± 10 V. Metabolite Pilot^TM^ (Sciex) software was used to establish a list of potential metabolites, using a set of known biotransformations (oxidative reactions, and GSH and NAC conjugates). Closely eluting isotope-labeled peaks were also searched, having from one to four deuterium labels in the final metabolite formula. Peakview/Masterview^TM^ (Sciex) software was then used for processing LC-MS/MS data, to validate the presence of all these metabolites. The fragment ions were proposed by rationally looking at parent compound spectra, high-resolution MS/MS spectra and proposed fragmentation from the literature. From the accurate mass MS/MS data, the molecular formula of fragment ions was determined, considering the starting formula of the precursor ion.

## 3. Results

**Figure 1:**
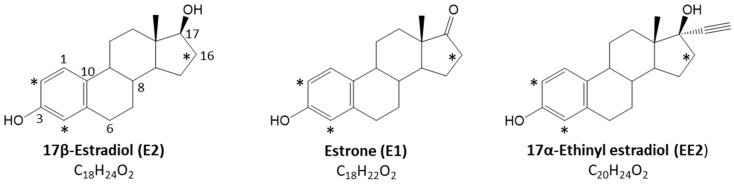
Chemical structures of the three estrogen analogs used in this study. Deuterated positions (2,4,16) are noted with * for d4-isotope-labeled standards used.

The metabolism of three estrogen analogs and their deuterated versions (Figure 1) was analyzed by LC-HRMS/MS to study the formation of oxidative metabolites in vitro. Two analogous soft nucleophiles, GSH and NAC, were used as trapping agents to detect unstable electrophilic species, by forming corresponding stable adducts via the nucleophilic attack of their free thiol groups. The high-resolution LC-MS profiles for each incubation type were compared to control samples to confirm the formation of metabolites and adducts.

**Figure 2:**
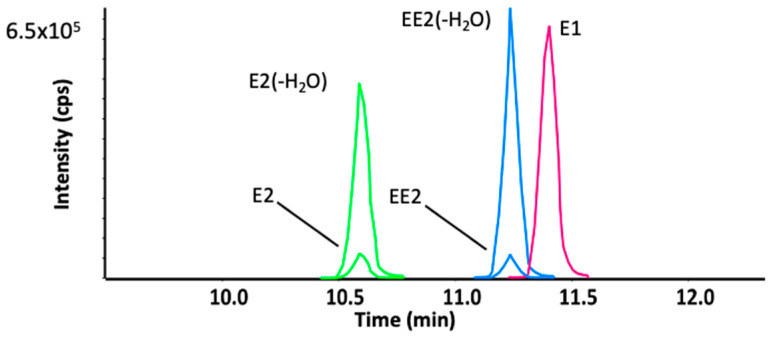
Extracted ion chromatograms for protonated E1, E2 and EE2 from control incubations (at 10 µM). Both E2 and EE2 exhibit a facile water loss in the electrospray source.

**Figure 3:**
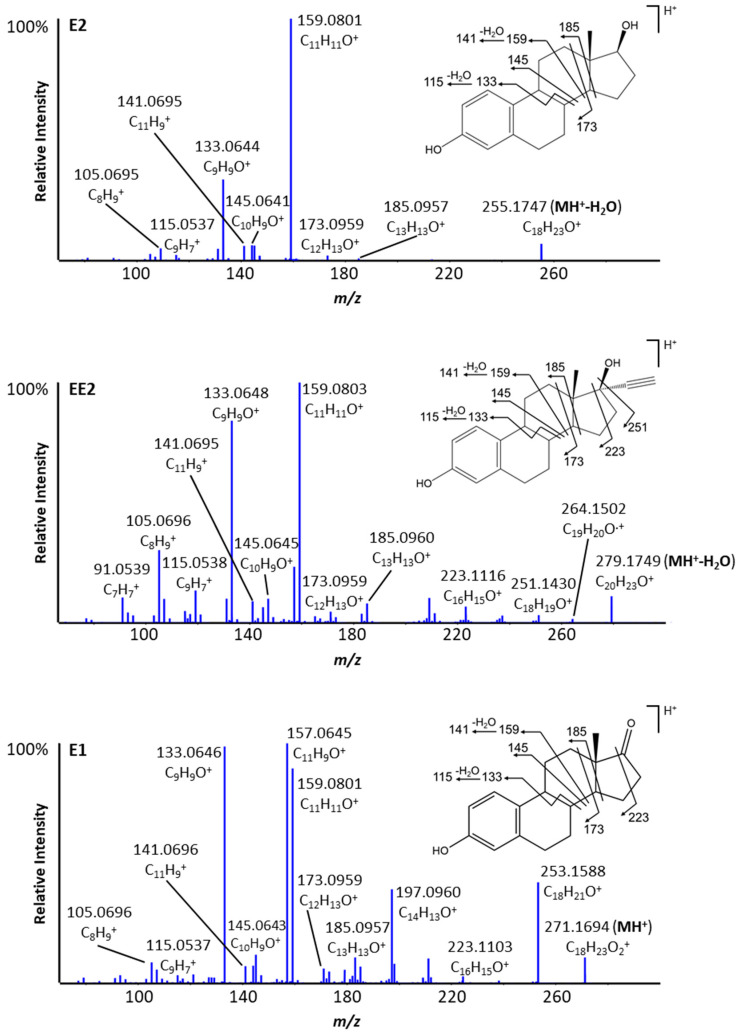
High-resolution MS/MS spectra of protonated ions of E2-H_2_O, EE2-H_2_O and E1 with annotated fragment ions, including mass accuracies (ppm).

Electrospray ionization mass spectra of E2 and EE2 (Figure 2) revealed that both hormones readily lose a water molecule in the electrospray source at carbon 17, with the dehydrated molecule becoming the predominant precursor ion detected. This in-source fragmentation does not occur in the case of E1, where a carbonyl group is present at C-17. The protonated ions of E2 (-H_2_O) (C_18_H_23_O^+^) and EE2 (-H_2_O) (C_20_H_23_O^+^) were detected at retention times of 10.6 and 11.2 min, respectively, under our chromatographic conditions. Protonated E1 (C_18_H_23_O_2_^+^) was detected at a retention time of 11.4 min. The fragmentation spectra from collision-induced dissociation indicate that all three hormones undergo similar fragmentation patterns. B, C and D rings cleave sequentially revealing characteristic fragment ions at *m*/*z* 133, 145, 159, 173 and 185 (Figure 3).

### 3.1. Oxidative Metabolites

**Figure 4:**
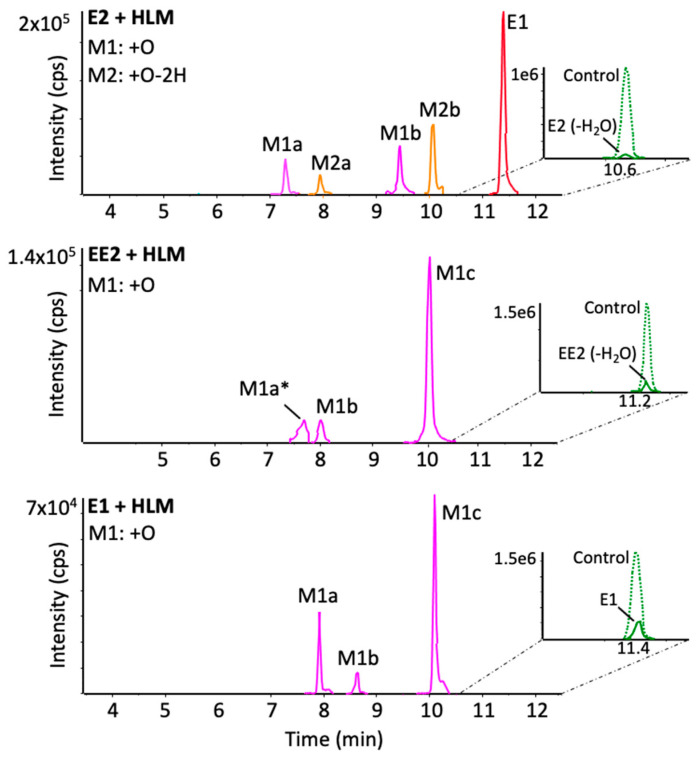
High-resolution extraction ion chromatograms of oxidative metabolites formed from E2, EE2 and E1 in human liver microsomal incubations. Some peaks (*) were increased 5× for clarity. Details of measured *m*/*z* and molecular formulae can be found in Table 1.

E2, E1 and EE2 undergo extensive metabolism in humans and animals. Numerous hydroxylated or ketone metabolites have been found, the formation of which is catalyzed by cytochromes P450. Hydroxylated metabolites detected in HLM are presented in Figure 4. Hydroxylation is known to occur at the 2- or 4-position of the A-ring, leading to the formation of catechol estrogens. Hydroxylated E2 and EE2 undergo in-source water loss as for the parent compounds. Two peaks, at 7.3 and 9.5 min, correspond to hydroxylated E2 metabolites (E2+O). In both cases, the fragment ion corresponding to C_11_H_11_O^+^ for E2 at *m*/*z* 159.080 is shifted to *m*/*z* 175.075 (Table S1), indicating that either A or B ring may be involved. However, analysis of the deuterated version of the two isomers indicates the loss of one deuterium (Table 1), meaning the A ring is hydroxylated (at position 2 or 4) and involved in both metabolites, forming 2-OH-E2 and 4-OH-E2. E2 can also form E1, and therefore many hydroxylated metabolites found in E1 incubations were also observed in E2 incubations corresponding to E2+O-2H (Figure 4) and E2+2O-2H (Figure S1). In the case of estrone, the two hydroxylated peaks (at 8 and 10 min, Figure 4) correspond to 2-OH-E1 and 4-OH-E1. Analysis of deuterated estrone incubations indicates the exchange of a deuterium in each case. A third hydroxylated metabolite corresponding to E1+O at 8.7 min (Figure 4) shows a fragmentation identical to E1. The fragment ion corresponding to C_16_H_17_O^+^ at *m*/*z* 225.127 is unchanged (Table S1), indicating that oxidation does not occur on the A-, B- or C-ring. In the deuterated metabolite, one label has been exchanged (Table 1), indicating that oxidation has occurred at carbon 16 of the D-ring, forming 16-OH-E1. As for EE2, the hydroxylated metabolite peak of highest intensity (at 10.1 min) corresponds to 2-OH-EE2 or 4-OH-EE2, with similar MS/MS behavior as mentioned above. At present, the literature only reports 2-OH-EE2 as the main metabolite formed by EE2 [21]. A smaller EE2+O metabolite peak (at 8.2 min) yields fragmentation like EE2 (Table S1) and suggests that metabolism is occurring on the D-ring; however, analysis of the labeled version indicates that no deuterium is exchanged. Together with the fact that the fragment ion corresponding to C_18_H_19_O^+^ in the parent is found unchanged in the MS/MS spectrum of the metabolite (at *m*/*z* 251.1431, Table S1), the oxidation occurs on the ethynyl group. Interestingly, this metabolite has never been reported in the literature.

**Figure 5:**
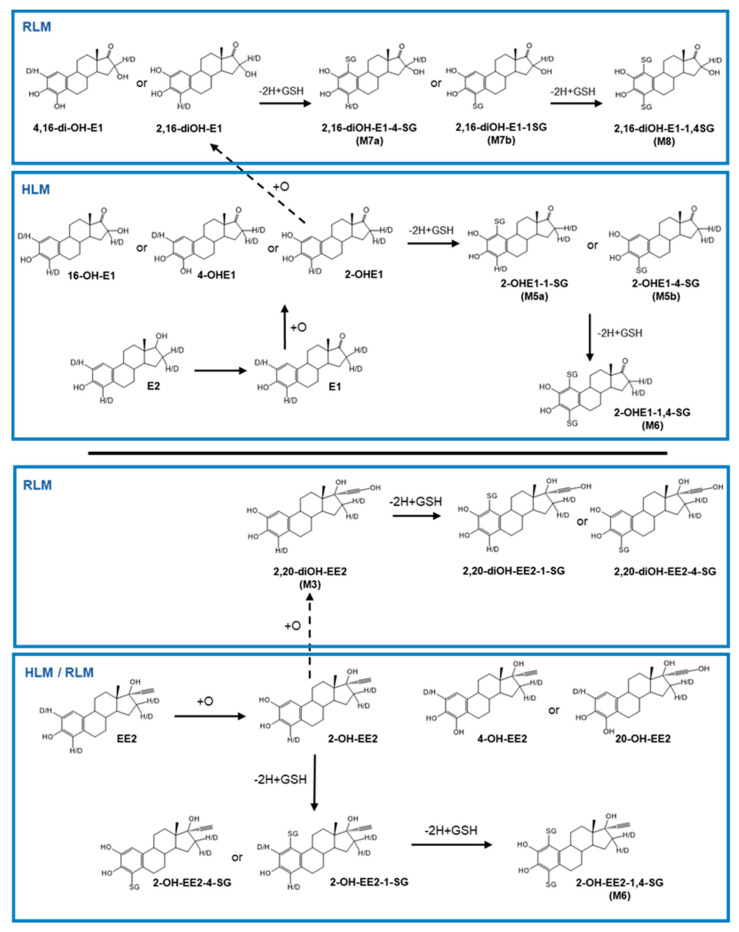
Proposed biotransformation reactions of unlabeled and deuterated E1, E2 and EE2 detected in human and rat microsomal incubations. As estrone and estradiol undergo similar metabolic profiles, only the structures from estrone are proposed to simplify the figure; the adduct formed with GSH can be formed with NAC. Peaks are annotated if structures could be determined for specific retention times.

When substituting human for rat liver microsomes, similar results were observed (Figure S1). However, some metabolites were uniquely observed in rat samples, for example dihydroxylated metabolites. For estrone, two peaks corresponding to E1+2O were observed at 4.8 and 7.4 min, with MS/MS fragmentation suggesting that oxidation has occurred on both the A- and D-ring. The fragment ions at *m*/*z* 133.065 and *m*/*z* 171.080, respectively, shifted to *m*/*z* 149.059 and *m*/*z* 187.075 (Table S1) indicating that the one oxidation occurs on the A-ring. This proposed structure is also supported by deuterated metabolites, showing an exchange of two labels (Table 1). The ion fragments at *m*/*z* 149.059 and *m*/*z* 187.075 were shifted to *m*/*z* 150.066 and *m*/*z* 188.079, indicating that one deuterium was displaced during the oxidation of ring A, at either position 2 or 4, with the second hydroxylation occurring at position 16. The same structure was observed for E2+2O, exhibiting an in-source water loss at 6.7 min. The data for EE2+2O (-H_2_O) (Figure S1) indicates that the first oxidation occurs at the A-ring for the two metabolites at 6.7 and 7.2 min. In fact, the ion fragment C_11_H_11_O^+^ at *m*/*z* 159.080 shifted to C_11_H_11_O_2_^+^ at *m*/*z* 175.075 (Table S1), and these same peaks are shifted to *m*/*z* 176.081, meaning that a deuterium was exchanged at the A-ring; however, the second oxidation would affect the D-ring without deuterium exchange. E2 undergoes a double hydroxylation (Figure S1); analysis of the MS/MS spectrum of the metabolite at 6.5 min indicates that the first hydroxylation is located at ring A, the fragment ion *m*/*z* 133.064 is displaced to *m*/*z* 149.060 (Table S1), while analysis of the deuterated version reveals that two deuterium atoms are exchanged (Table 1). The first deuterium exchanged would be located on ring A, as the fragment ion *m*/*z* 149.060 is displaced to *m*/*z* 150.066, indicating that only one deuterium is displaced on ring A, which implies that the second oxidation is located on the D-ring, displacing a deuterium in position 16. The proposed structures for each of the major oxidative metabolites formed during with HLM and RLM are shown in Figure 5. As estrone and estradiol undergo the same metabolic pathways, only the structures of the metabolites obtained from estrone are shown to simplify the figure.

### 3.2. Reactive Metabolites

Xenobiotics can be metabolized into stable oxidized metabolites to allow their elimination from the body. However, some can also form reactive metabolites, which are unstable electrophiles with the potential to alter endogenous macromolecules, such as proteins and DNA, inducing hepatotoxicity and genotoxicity [22]. This is one of the many routes by which drugs can exhibit toxicity. Due to their instability, direct detection and structural characterization of reactive metabolites are inherently difficult. They are classified as either soft or hard reactive metabolites, based on their polarizability. Soft reactive metabolites include electrophilic metabolites such as quinones, imine quinones and epoxides [23]. These metabolites react readily with the sulfhydryl group of cysteine or glutathione. By contrast, hard reactive metabolites, such as aldehydes, preferentially react with the amine groups, such as lysine, arginine, and nucleic acids [17]. A commonly used approach for assessing the formation of reactive metabolites is to trap them with a nucleophilic compound, resulting in stable adducts, which can then be characterized.

#### 3.2.1. GSH and NAC Adducts

**Figure 6:**
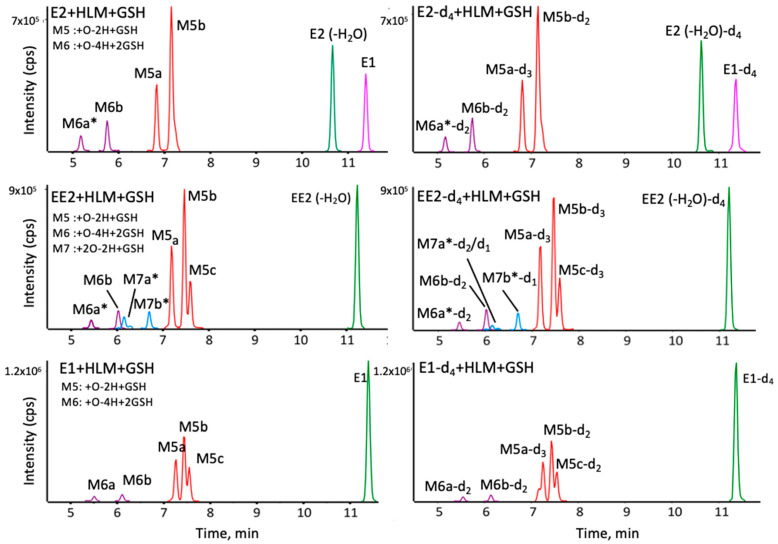
Extracted ion chromatograms of E2, EE2 and E1 metabolites and adducts trapped by GSH, for their non-labeled (left) and deuterated versions (right). Some peaks (*) were increased 5× for clarity. Details of measured *m*/*z* and molecular formulae can be found in Table 1.

Glutathione (GSH) is commonly used as a scavenging molecule in microsomal incubations to trap soft reactive electrophilic metabolites, similar to how it scavenges reactive oxygen species in the body as a defense mechanism against oxidative stress. *N*-acetylcysteine is also used as a soft reactive metabolite scavenger, with the same free thiol group as GSH, and comparing results from both trapping agents helps confirm the results. When E1, E2 and EE2 were incubated under oxidative conditions in the presence of GSH or NAC, several analogous single or double adducts were detected, as shown in Figure 6. NAC adducts showed increased retention on the chromatographic column. For instance, the intense peak corresponding to E2+O-2H+GSH at 7.2 min (Figure 6) and an analogous signal for E2+O-2H+NAC at 8.8 min (Figure S2). This difference could allow the detection of adducts of more polar reactive metabolites for NAC compared to GSH. In addition, NAC trapping allowed the detection of an additional isomer of +O-2H + NAC in E2, most likely from the ability to separate these two isomers of NAC adducts, with the two analogous GSH adducts likely co-eluting (Figure S2).

#### 3.2.2. Deuterated Versions of Trapped Adducts

**Figure 7:**
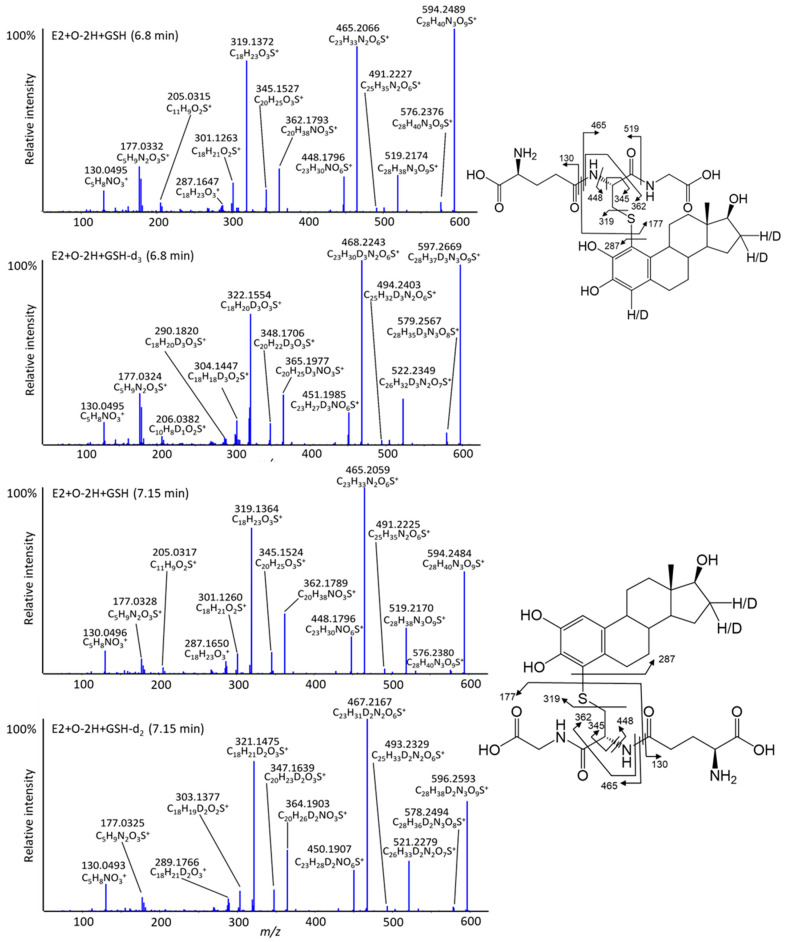
High-resolution MS/MS spectra of isomeric E2+O-2H+GSH adducts in their unlabeled and labeled forms, all fragment ions assigned were under 5 ppm.

To help confirm the specific sites involved in the metabolism of these compounds, deuterated versions of each were incubated under the same conditions. As can be seen in Figure 5, labeled compounds exhibit the same retention and with similar intensities as their non-deuterated versions (Figure 6). For example, the two GSH adducts of E2 (at 6.8 and 7.2 min, with an *m*/*z* of 594.248, Figure 7) can be confirmed using the deuterated versions. The MS/MS spectra of these isomers are dominated by the characteristic fragment ions of GSH, generally observed in positive ion mode at *m*/*z* 233 and 179 via neutral losses of glycine 75 D and pyroglutamic acid 129 Da, respectively [24]. For the 6.8 min isomer, E2-d_4_ exchanges one deuterium (Figure 7) upon oxidation to form an ortho-quinone in position 2. GSH is then added at position 1 as shown in the proposed structure. The isomer at 7.2 min exchanges two labels, indicating a loss of deuterium in position 1, forming the ortho-quinone, and the addition of GSH in position 4. Another interesting peak appears at 6 min, corresponding to +O-4H+2GSH, with the loss of two labels (Table 1), implying that a second oxidation occurs at position 2 forming the ortho-quinone and that two GSH molecules are placed in positions 1 and 4. In RLM incubations (Figure S3), two previously unreported adducts were detected involving double hydroxylation and addition of one or two GSH molecules, forming E+2O-2H+GSH and E+2O-4H+2GSH. When incubated with HLM, only EE2 produced the double hydroxylated adduct EE2+2O-2H+GSH. The MS/MS spectra of these different metabolites are dominated by specific fragments of the trapping agent in contrast to the parent hormones. It is therefore difficult to locate the adduction or hydroxylation sites. However, analysis of the deuterated analogs provides more information (Table 1). For EE2, for example, a peak is observed (Figure 6) corresponding to EE2+2O-2H+GSH at 6.2 min with an *m*/*z* 634.242 (Table 1). The deuterated version of this adduct shows an exchange of two labels at *m*/*z* 636.253, which suggests that the double hydroxylation and adduction occurred on ring A. A first hydroxylation in position 2 could have led to the formation of ortho-quinone trapped by GSH in position 4, the second hydroxylation would then have occurred in position 1 or another ring without inducing deuterium exchange. When incubated with RLM, E2 can form E2+2O-4H+2GSH at *m*/*z* 458.160, implying double hydroxylation and double adduction. Analysis of the MS/MS spectrum of the deuterated version indicates an exchange of three labels yielding a precursor ion with *m*/*z* 458.662 at 5.1 min (Table 1). This could be explained by a first hydroxylation at position 2 forming the orthoquinone where then GSH attacks at position 4, followed by a subsequent re-formation of o-quinone and a second GSH added at position 1. The second hydroxylation site is at position 16, displacing a deuterium.

When the three analogs and their deuterated versions were incubated in the presence of NAC (Figure S2), similar results to those obtained with GSH were observed. NAC and GSH should form very similar adducts, and the deuterated versions of these adducts confirm this, as the same number of labels are displaced for each adduct formed. For example, the E2+O-2H+NAC isomers at 8.0 and 8.6 min (Figure S2) have similar structures as their GSH analogs. Although the MS/MS spectra of the NAC adducts are dominated by the fragmentation of the scavenger (as for GSH), it was possible to demonstrate that the hydroxylation and adduction of NAC are localized on the A-ring. Indeed, the two isomers both form a fragment at *m*/*z* 173.059 (Table S1), corresponding to C_11_H_9_O_2_. This indicates that the hydroxylation could be located on the A or B-ring (Figure 3). Studying the labeled compounds and their metabolites allows a more precise determination of the hydroxylation and adduction sites. In the case of E2, the use of NAC allowed the identification of a third E2+O-2H+NAC isomer at 8.8 min, whose GSH analog could not be separated chromatographically. Analysis of the deuterated versions indicates that two labels are lost for two adducts and only one is lost in the case of one adduct. Therefore, hydroxylation occurs at position 2 with NAC addition at position 4, or vice versa, for the two possible isomers losing two labels.

The proposed structures for each of the major adducts formed during microsomal incubations from both species are shown in Figure 5. As estrone and estradiol undergo similar metabolic profiles, only the structures from estrone and adducts with GSH are proposed to simplify the figure.

## 4. Discussion

### 4.1. Oxidative Metabolism

The endogenous estrogens E2 and E1, as well as the synthetic derivative EE2, are known to be highly metabolized in human and various animal models. The resulting metabolites can play a key role in developmental processes but can also exhibit negative side effects. High-resolution tandem mass spectrometry was used to detect and characterize the reactive metabolites capable of forming GSH and NAC adducts in vitro. In addition, an isotope-labeled version for each analog was used to facilitate the structural elucidation of metabolites and adducts. Suchar et al. demonstrated that incubation of radiolabeled E2 in the presence of rat and mouse liver microsomes produced 15 metabolites [11,19]. Lee et al. used HPLC-UV and radioactivity to characterize the NADPH-dependent metabolism profile of E2 and E1 in human liver microsomes, with many hydroxylated or ketone metabolites being identified [25]. Estrone and estradiol are rapidly interconverted in the body by the enzymes 17beta-hydroxysteroid dehydrogenase type 1 and 2, catalyzing the reduction or oxidation of the ketone or hydroxyl found at position 17 [26]. The main metabolite formed by these two hormones is 2-hydroxy estrogen (2-OHE) followed by 4-hydroxy estrogen (4-OHE), forming catechol estrogens corresponding to the oxidative metabolites detected for each analog (Figure 4). However, 4-OH-E2 and 16-OH-E1 have been shown to exhibit high genotoxicity [27], and 4-OH-E2 is considered the main metabolite involved in estrogen-dependent cancers. It has been shown that 17α-ethinyl estradiol is associated with an increased risk of developing breast, uterine and liver cancer. Wan et al. have shown the molecular mechanisms of liver damage involve the oxidation of EE2. EE2 undergoes the same P450 isoform-induced metabolism as its analogs (Table 1). It has been shown that 2-OH-EE2 is the main oxidative metabolite. However, in our study, we were able to identify a new oxidation site for EE2, in the ethynyl group. It is suggested that EE2, unlike E2 and E1, is less likely to undergo oxidation at the C16 position due to steric hindrance, and the isotopically labeled experiment confirms this. The oxidation occurs instead at the more accessible C20.

### 4.2. GSH et NAC Adducts

Similar reactive metabolites were seen to be scavenged by GSH and NAC, in the presence of HLM and RLM. Previous studies have shown that oxidation of estrogens by P450 isoforms results in the formation of catechol estrogens, which can subsequently generate reactive ortho-quinones and be trapped by GSH. The ortho-quinone 2-OHE1/E2/EE2 is thought to be more reactive than the 4-OHE1/E2/EE2 and more likely to bind GSH and NAC, forming trapped species +O-2H+GSH (Figure 6) and +O-2H+NAC (Figure S2). It has been shown that quinones formed by the oxidation of estrogenic catechol undergo redox cycling, allowing them to react with cellular nucleophiles, such as DNA, protein and endogenous glutathione. The binding of GSH to quinones allows their elimination, thus acting as a protective mechanism. However, the binding of quinones to hepatic GSH appears to lead to the formation of quinine thioethers which are structurally like M4 (+O-2H+GSH), and these have potent nephrotoxic properties. Butterworth et al. demonstrated that E2 catecholate estrogens cause acute nephrotoxicity in hamsters by inhibiting γ-glutamyl transpeptidase (an enzyme involved in amino acid metabolism), the substrate for quinone thioethers [19]. In the presence of RLM, two additional adducts were detected, with double hydroxylation and double adduction. In a previous study, Iverson et al. investigated the factors influencing the rate of P450-catalysed formation of o-quinones from 2-OH-E1 and 4-OH-E1 and estrone in rat liver microsomes [8]. GSH was used to trap the quinones and they were separated and characterized by HPLC-MS. As indicated by our results, mono-GSH conjugates were observed to be formed from 2-OH-E1-o-quinone corresponding. The 2-OH-E1-o-quinone appears to also form a di-GSH conjugate corresponding to E1+O-4H+2GSH. O-quinones are also responsible for the induction of oxidative DNA damage by estrogen catechol; by alkylating DNA and thus inducing irreversible damage, 4-OH-E1-o-quinone modifies adenine at the C8 position on the purine ring under reducing conditions [28]. In hamsters, 4-OH-E2 was shown to be responsible for the development of kidney tumors, by reacting with DNA to form a depurative adduct (leaving apurinic sites on the DNA) [29].

## 5. Conclusions

Liquid chromatography coupled to high-resolution mass spectrometry was used to detect and characterize the oxidative metabolites of three estrogen analogs (E2, E1 and EE2). Two different trapping agents were employed to trap soft electrophiles formed following oxidation of parent compounds as stable adducts. Detailed MS/MS fragmentation pathways, aided by isotope labeling, were used to elucidate the structure of metabolites. Deuterated compounds are specifically interesting to use in the context of metabolism studies since labels can be lost during metabolism. The sites of deuterium labeling were specifically helpful in pinpointing sites of metabolism and confirming resulting structures. Combining unlabeled and labeled compounds with high-resolution mass spectrometry data is beneficial to confirming which compounds are metabolites of the studied parent compounds, in complex biological samples with many endogenous metabolites having similar chemical formulae. This approach could be used to support studies to identify the metabolites of other estrogen analogs or similar xenobiotics. Many new metabolites and adducts were found for the first time, including those involving di-hydroxylation, as well as NAC adducts. The biological relevance of the formation of these metabolites would necessitate further in vivo studies.

## Figures and Tables

**Table 1:** Summary of metabolites and adducts characterized for E1, E2 and EE2 in human and rat liver microsomes.

Biotransformation	Formula	RT (min)	*m*/*z* (*ppm*)	Δm(d_4)_
**Estrone**	C_18_H_22_O_2_	11.4	271.1689 (–1.4)	4
+O	C_18_H_22_O_3_	8.0	287.1641(–0.3)	3
		8.7	287.1642 (–0.6)	3
		10	287.164 (–0.6)	3
+2O ^b^	C_18_H_22_O_4_	4.8 *	303.1584 (–2.1)	2
		7.4 *	303.1583 (–2.7)	2
+O-2H+GSH	C_28_H_37_N_3_O_9_S	7.25	592.2309 (–2.4)	3
		7.4	592.2307 (–2.8)	2
		7.6	592.2311 (–2.1)	2
+2O-2H+GSH ^b^	C_28_H_37_N_3_O_10_S	6 *	608.2278 (0.9)	1
		6.4 *	608.228 (1.2)	2
+O-4H+2GSH	C_38_H_52_N_6_O_15_S_2_	5.3 *	449.155^+2^ (2.4)	2
		5.5	449.1542^+2^ (0.8)	2
		6.2	449.154^+2^ (0.3)	2
+2O-4H+2GSH ^b^	C_38_H_52_N_6_O_16_S_2_	5.6 *	457.152^+2^ (1.1)	1
+O-2H+NAC ^b^	C_23_H_29_NO_6_S	8.7	448.1791 (0.5)	3
		9.1	448.1789 (0.2)	2
		9.3	448.1786 (–0.6)	2
+O-4H+2NAC ^b^	C_28_H_36_N_2_O_9_S_2_	7.4	609.193 (–0.8)	2
		8.1	609.193 (–0.9)	2
**ß-Estradiol**	C_18_H_24_O_2_	10.7	273.185 (0.5)	4
ß-Estradiol	C_18_H_22_O	10.7	255.1746 ^a^ (0.9)	4
-2H (E1)	C_18_H_22_O_2_	11.4	271.1694 (0.4)	4
+O	C_18_H_22_O_2_	7.3	271.1693 ^a^ (0.5)	3
		9.5	271.1693 ^a^ (0.3)	3
+O-2H	C_18_H_22_O_3_	8	287.1644 (0.7)	3
		10	287.1644 (0.6)	3
+2O-2H ^b^	C_18_H_22_O_4_	4.8 *	303.1601 (3.3)	2
		7.4 *	303.1591 (0.7)	2
+2O ^b^	C_18_H_22_O_3_	6.5 *	287.1647 ^a^ (1.7)	2
+O-2H+GSH	C_28_H_39_N_3_O_9_S	6.8	594.2486 (1.0)	3
		7.15	594.2488 (1.4)	2
+2O-2H+GSH ^b^	C_28_H_39_N_3_O_10_S	5.6 *	610.2426 (–0.5)	1
		5.9 *	610.2435 (1.0)	2
+O-4H+2GSH	C_38_H_54_N_6_O_15_S_2_	5.2	450.1626^+2^ (2.0)	2
		5.75	450.1637^+2^ (2.3)	2
+2O-4H+2GSH ^b^	C_38_H_54_N_6_O_16_S_2_	5.1 *	458.1608^+2^ (1.3)	1
		5.4 *	458.1608^+2^ (1.5)	2
+O-2H+NAC ^b^	C_23_H_31_NO_6_S	8.0	450.1951 (1.4)	3
		8.6	450.1951 (1.3)	2
		8.8	450.1941 (–0.9)	2
+O-4H+2NAC ^b^	C_28_H_38_N_2_O_9_S_2_	6.8	611.2106 (2.3)	2
		7.4	611.2092 (0.1)	2
**Ethinyl estradiol**	C_20_H_24_O_2_	11.2	297.1847 (–0.7)	4
**EE2(-H_2_O)**	C_20_H_20_O	11.2	279.1744 (0.2)	4
+O	C_20_H_22_O_2_	7.8	295.1698 ^a^ (1.7)	4
		8.2	295.1695 ^a^ (0.9)	4
		10.1	295.1695 ^a^ (0.8)	3
+2O ^b^	C_20_H_22_O_3_	6.7 *	311.1644 ^a^ (0.6)	3
		7.2 *	311.1645 ^a^ (1.1)	3
+O-2H+GSH ^b^	C_30_H_39_N_3_O_9_S	7.2	618.2492 (1.9)	3
		7.5	618.2477 (–0.4)	2
		7.7	618.2478 (–0.2)	2
+2O-2H+GSH ^b^	C_30_H_39_N_3_O_10_S	6.15	634.2418 (–1.7)	2/1
		6.3 *	634.2421 (–1.2)	2
		6.5 *	634.2423 (–0.9)	3
		6.7	634.2423 (–1.0)	3
+O-4H+2GSH ^b^	C_40_H_54_N_6_O_15_S_2_	5.1 *	462.1624^+2^ (1.5)	2
		5.5	462.162^+2^ (0.7)	2
		6	462.1622^+2^ (1.2)	2
+O-2H+NAC ^b^	C_25_H_31_NO_6_S	8.55	474.1940 (–1.0)	3
		9.05	474.1947 (0.3)	2
		9.3	474.1945 (0.0)	2
+2O-2H+NAC ^b^	C_25_H_31_NO_7_S	8.3	490.1885 (–1.9)	2
+O-4H+2NAC ^b^	C_30_H_38_N_2_O_9_S_2_	7.3	635.2085 (–1.1)	2
		7.9	635.2086 (–0.9)	2

* Only detected in RLM, ^a^ *m*/*z* from in-source fragment from water loss, ^b^ metabolites and adducts reported for the first time.

## Data Availability

Data are contained within the article and Supplementary Materials.

## Supplementary Materials

The following are available online at https://www.mdpi.com/article/10.3390/metabo12100931/s1, Figure S1. High-resolution extraction ion chromatograms of oxidative metabolites formed from E2, EE2 and E1 in rat liver microsomal incubations. Figure S2. Extracted ion chromatograms of E2, EE2 and E1 metabolites and adducts trapped by NAC, for their non-labeled and deuterated versions. Figure S3. Extracted ion chromatograms of E2, EE2 and E1 metabolites and adducts trapped by GSH. Table S1. Summary of metabolites and adducts characterized for E1, E2 and EE2 in human and rat liver microsomes.
