# Supplementary material for: Estradiol, Estrone and Ethinyl Estradiol Metabolism Studied by High Resolution LC-MS/MS Using Stable Isotope Labeling and Trapping of Reactive Metabolites
Source: Metabolites. 2022 Sep 30;12(10):931. doi: 10.3390/metabo12100931 (PMC9611524; doi:10.3390/metabo12100931)
Supplement: Supplementary file 1 [file metabolites-12-00931-s001.zip › metabolites-1911713-supplementary.pdf]

metabolites

MDPI

Supplemental Material

Estradiol, estrone and ethinyl-estradiol metabolism studied by high resolution LC-MS/MS using stable isotope labeling and trapping of reactive metabolites

Kahina Chabi , Lekha Sleno *

Figure S1. High-resolution extraction ion chromatograms of oxidative metabolites formed from E2, EE2 and E1 in rat liver microsomal incubations.

Figure S2. Extracted ion chromatograms of E2,EE2 and E1 metabolites and adducts trapped by NAC, for their non-labeled and deuterated versions. Some peaks (*) were increased by 5× for clarity.

Figure S3. Extracted ion chromatograms of E2,EE2 and E1 metabolites and adducts trapped by GSH.

Table S1. Summary of metabolites and adducts characterized for E1, E2 and EE2 in human and rat liver microsomes, fragments related to the trapping agent are shown in bold.

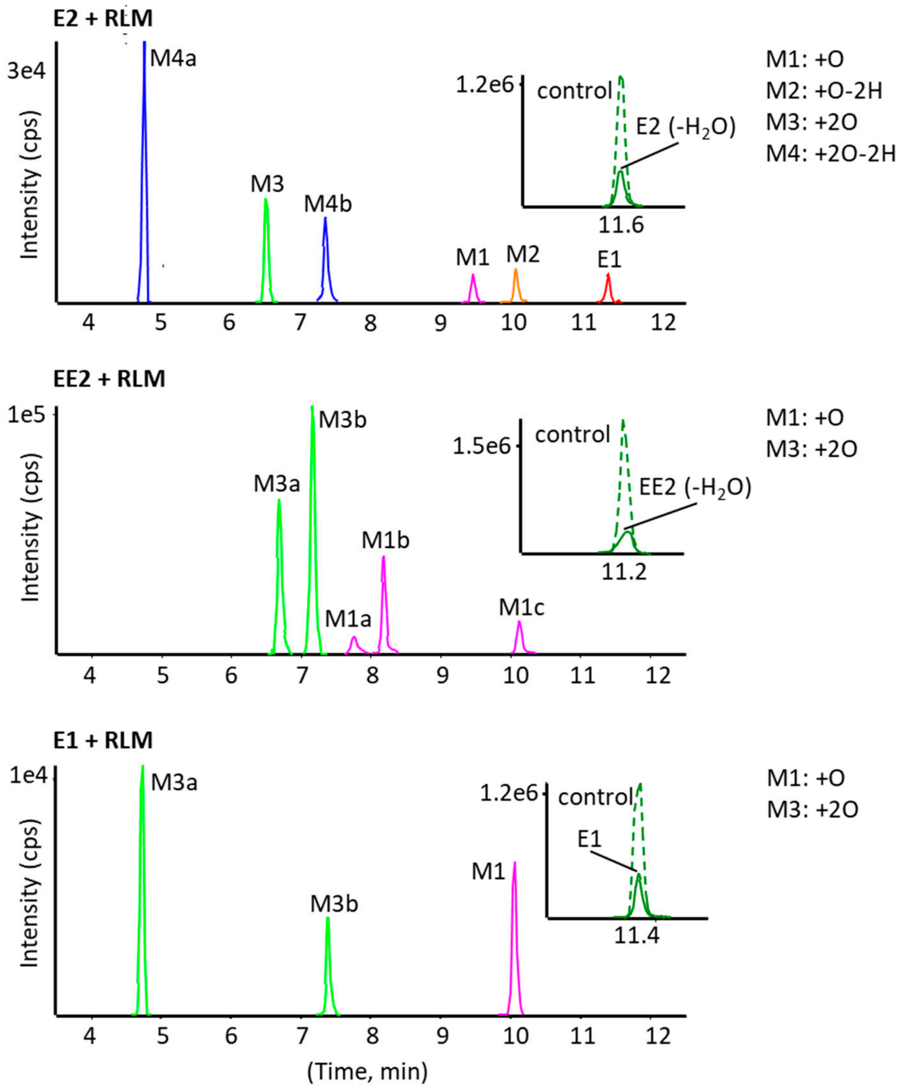

Figure S1. High-resolution extraction ion chromatograms of oxidative metabolites formed from E2, EE2 and E1 in rat liver microsomal incubations.

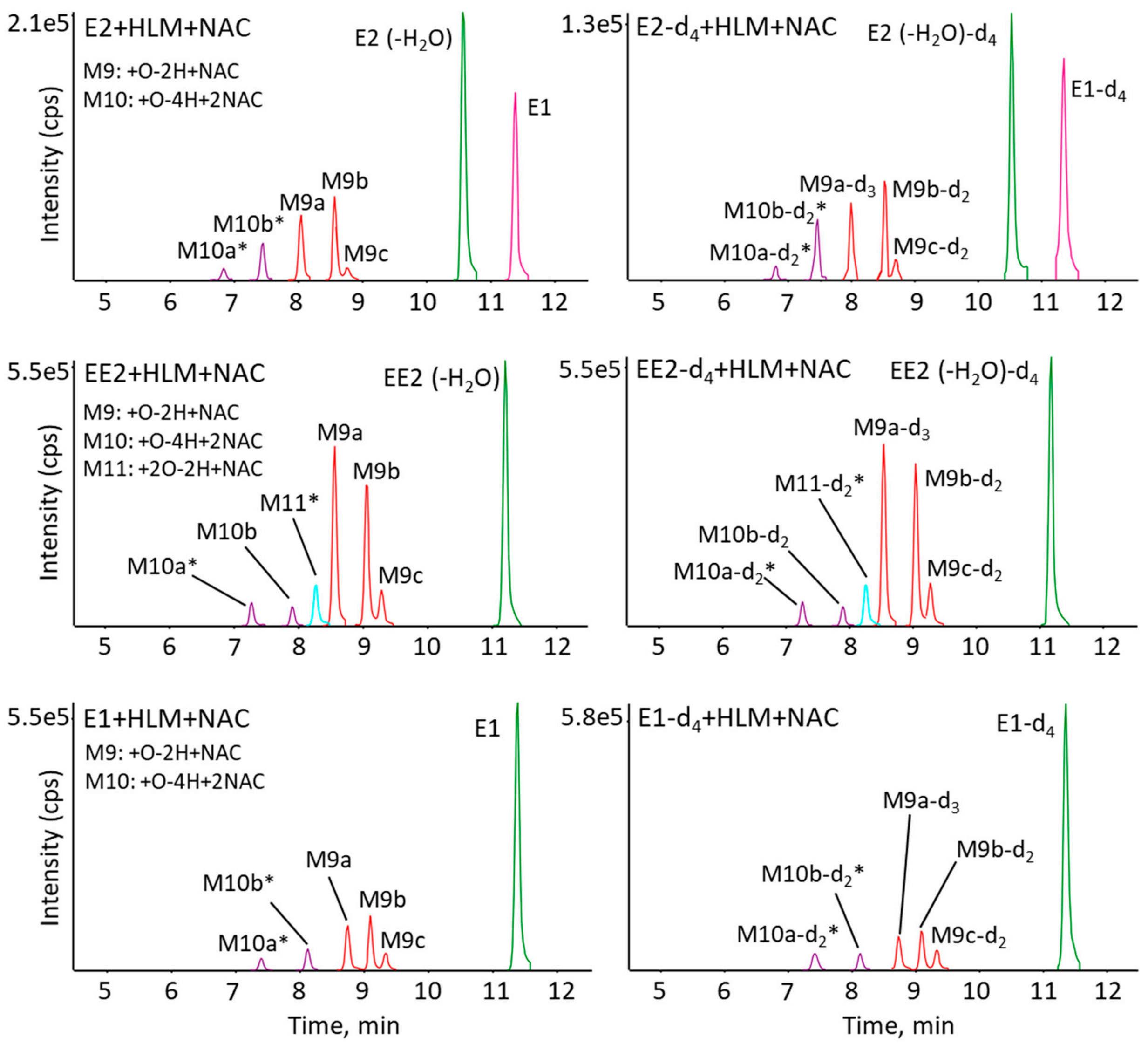

Figure S2. Extracted ion chromatograms of E2,EE2 and E1 metabolites and adducts trapped by NAC, for their non-labeled and deuterated versions. Some peaks (*) were increased by 5× for clarity.

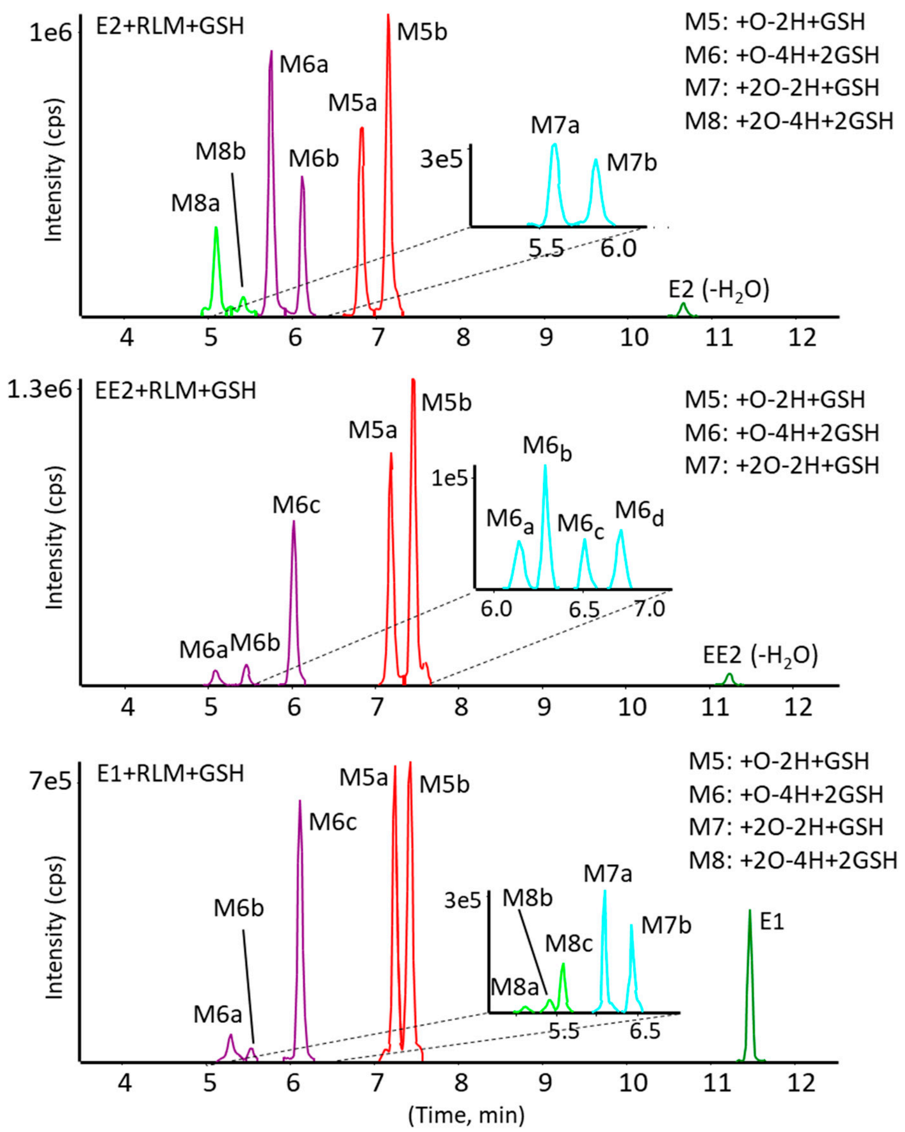

Figure S3. Extracted ion chromatograms of E2,EE2 and E1 metabolites and adducts trapped by GSH.

Table S1. Summary of metabolites and adducts characterized for E1, E2 and EE2 in human and rat liver microsomes, fragments related to the trapping agent are shown in bold. (continued).

Biotransformation	Formula	RT (min)	Measured *m/z* (ppm)	Selected MS/MS fragments
Estrone (parent)	$C_{18}H_{22}O_2$	11.4	271.1689 (-1.4)	253.1588, 223.1103, 197.0960, 185.0957, 173.0959, 159.0801, 157.0645, 145.0643,141.0696, 133.0646
+O	$C_{18}H_{22}O_3$	8	287.1641 (-0.3)	269.1536, 213.0909, 199.0756, 175.0748
		8.7	287.16417 (-0.6)	269.1543, 251.1433, 225.1281, 213.1269, 199.1118, 159.0804, 133.0655
		10	287.1640 (-0.6)	269.1531, 229.1218, 213.0907, 199.0750, 161.0592
+2O	$C_{18}H_{22}O_4$	4.8*	303.1584 (-2.1)	285.1479, 241.1227, 227.1067, 211.0763, 201.0914, 175.0744, 173.0591, 149.0592
		7.4*	303.1583 (-2.7)	285.1490, 267.137, 215.1064, 201.0914, 187.07531, 175.0744, 173.0591, 149.0592
+O-2H+GSH	$C_{28}H_{37}N_3O_9S$	7.25	592.2309 (-2.4)	**592.2308, 517.1980, 463.1886, 446.1613, 360.1614, 317.1199, 177.0321**
		7.4	592.2307 (-2.8)	**517.1988, 463.1880, 446.1620, 360.1621, 317.12, 177.0325, 130.0495**
		7.6	592.2311 (-2.1)	**592.2311, 517.1980, 463.1887, 446.1620, 360.1606, 317.1198,130.0493**
+2O-2H+GSH	$C_{28}H_{37}N_3O_{10}S$	6*	608.2278 (0.9)	**608.2278, 590.2174, 533.1953, 479.1851, 462.1581, 376.1576, 333.1153, 177.0320, 130.0494**
		6.4*	608.2280 (1.2)	**608.2278, 533.1954, 479.1850, 462.1571, 376.1582, 333.1158, 177.0320, 130.0495**
+O-4H+2GSH	$C_{38}H_{52}N_6O_{15}S_2$	5.3*	449.1550^{+2} (2.4)	**590.2176, 515.1816, 461.1743, 308.0910**, 233.0594, **179.0485, 162.0216, 130.0494**
		5.5	449.1542^{+2} (0.8)	**622.1881, 606.1981, 590.2190, 549.1731, 515.1746, 495.1565, 461.1686, 443.1637, 177.0298, 130.0500**
		6.2	449.1540^{+2} (0.3)	**622.1859, 493.1454, 384.6317, 177.0327, 130.0496**
+2O-4H+2GSH	$C_{38}H_{52}N_6O_{16}S_2$	5.6*	457.1520^{+2} (1.1)	**590.2177, 515.1882, 177.0330**
+O-2H+NAC	$C_{23}H_{29}NO_6S$	8.7	448.1791 (0.5)	**343.1359, 162.0213**
		9.1	448.1789 (0.2)	**406.1671, 343.1364, 162.0216, 130.0492**
		9.3	448.1786 (-0.6)	**406.1673, 343.136, 162.0214, 130.0490**
		7.4	609.1930 (-0.8)	**448.1788, 404.1501, 343.1403, 162.0216**
+O-4H+2NAC	$C_{28}H_{36}N_2O_9S_2$	8.1	609.1930 (-0.9)	**591.1823, 504.1518, 448.1780,** 285.1483, **162.0216**

Table S1. Summary of metabolites and adducts characterized for E1, E2 and EE2 in human and rat liver microsomes, fragments related to the trapping agent are shown in bold. (continued).

Biotransformation	Formula	RT (min)	Measured *m/z* (ppm)	Selected MS/MS fragments
ß-Estradiol	$C_{18}H_{24}O_2$	10.7	273.1850 (0.5)	251.1422, 197.0962, 159.0808, 147.0797
ß-Estradiol	$C_{18}H_{22}O$	10.7	255.1746 (-H_2O) (0.9)	199.1117, 185.0957, 173.0959, 159.0801, 145.0643, 141.0695, 133.0644
-2H (E1)	$C_{18}H_{22}O_2$	11.4	271.1694 (0.4)	213.1274, 197.0960, 183.0801, 171.0801, 159.0801, 157.0645, 145.0643, 133.0646
+O	$C_{18}H_{22}O_2$	7.3	271.1693(-H_2O) (0.5)	271.1697, 175.0748, 149.0589
		9.5	271.1693 (-H_2O) (0.3)	271.1696, 175.0746, 161.0593
+O-2H	$C_{18}H_{22}O_3$	8	287.1644 (0.7)	269.1536, 213.0909, 199.0756, 175.0748
		10	287.1644 (0.6)	269.1531, 229.1218, 213.0907, 199.0750, 161.0592
+2O-2H	$C_{18}H_{22}O_4$	4.8*	303.1601 (3.3)	285.1479, 241.1227, 227.1067, 211.0763, 201.0914, 175.0744, 173.0591, 149.0592
		7.4*	303.15909 (0.7)	285.1490, 267.137, 215.1064, 201.0914, 175.0744, 173.0591, 149.0592
+2O	$C_{18}H_{22}O_3$	6.5*	287.1647 (-H_2O) (1.7)	269.1544, 243.1389, 227.1066, 187.0756, 175.0760, 173.0597, 149.0598
+O-2H+GSH	$C_{28}H_{39}N_3O_9S$	6.8	594.2486 (1)	**594.2488, 576.2376, 519.2174, 465.2066, 319.1372, 301.1263**
		7.15	594.2488 (1.4)	**448.1796, 362.1792, 177.0331, 130.0494**
+2O-2H+GSH	$C_{28}H_{39}N_3O_{10}S$	5.6*	610.2426 (-0.5)	**519.2169, 465.2058, 448.1795, 362.1789, 319.1363, 301.1260, 177.0327, 130.0496**
		5.9*	610.2435 (1)	**535.2105, 481.2000, 464.1736, 378.1735, 335.1310, 317.1208, 177.0323, 130.0496**
+O-4H+2GSH	$C_{38}H_{54}N_6O_{15}S_2$	5.2	450.1626^{+2} (2)	**610.2441, 535.2120, 481.2012, 464.1748, 378.1738, 335.1317, 177.0325, 130.0492**
		5.75	450.1637^{+2} (2.3)	**594.2550, 465.2081, 441.1526, 319.1351, 130.0492**
+2O-4H+2GSH	$C_{38}H_{54}N_6O_{16}S_2$	5.1*	458.1608^{+2} (1.3)	**624.2043, 606.1940,594.2486, 465.2013, 549.1765, 495.1604, 177.0334**
		5.4*	458.1608^{+2} (1.5)	**565.1653, 511.1562, 177.0326, 130.0495**
+O-2H+NAC	$C_{23}H_{31}NO_6S$	8	450.1951(1.4)	**592.2262, 517.1979, 446.1625, 345.1478, 177.0322**
		8.6	450.1951 (1.3)	**345.1513, 319.1366,** 287.1638, 269.1538, **164.0369**
		8.8	450.1941 (-0.9)	**408.1858, 345.1529, 319.1351,** 287.1639, 269.1527, **162.0214**
+O-4H+2NAC	$C_{28}H_{38}N_2O_9S_2$	6.8	611.2106 (2.3)	**408.1859, 345.1527, 319.1351,** 287.1640, 269.1527, **162.0212**
		7.4	611.2092 (0.1)	**593.2007, 162.0217, 130.0497**

Table S1. Summary of metabolites and adducts characterized for E1, E2 and EE2 in human and rat liver microsomes, fragments related to the trapping agent are shown in bold. (continued).

Biotransformation	Formula	RT (min)	Measured *m/z* (ppm)	Selected MS/MS fragments
**Ethinyl-estradiol**	$C_{20}H_{24}O_2$	11.2	297.1847 (-0.7)	185.0956, 145.0638
**EE2**	$C_{20}H_{20}O$	11.2	279.1744 (-H₂O) (0.2)	264.1502, 251.1430, 223.1116, 185.0960, 173.0959, 159.0803, 145.0645, 141.0695 133.0648
+O	$C_{20}H_{22}O_2$	7.8	295.1698(-H_2O) (1.7)	277.1599, 251.1433, 235.1129, 197.0959, 171.0802, 159.0798
		8.2	295.1695(-H2O) (0.9)	277.1587, 251.1431, 235.1109, 227.1425, 211.1118, 197.0956, 185.0961, 171.0805, 159.0799, 145.0642
		10.1	295.1695(-H2O) (0.8)	251.1429, 225.0900, 213.0908, 187.0747, 175.0748, 149.0589
+2O	C20H22O3	6.7*	311.1644 (-H2O) (0.6)	293.1523, 265.1220, 251.1431, 215.1065, 201.0911, 187.0749, 175.07452, 173.0592
		7.2*	311.1645 (-H2O) (1.1)	293.1532, 265.1216, 251.1435, 215.1059, 201.0901, 187.0751, 175.0748, 173.0592,
+O-2H+GSH	$C_{30}H_{39}N_3O_9S$	7.2	618.2492 (1.9)	**543.216, 489.2055, 472.1793, 386.1788, 177.0324, 343.1363, 130.0492**
		7.5	618.2477 (-0.4)	**543.2152, 489.2047, 472.1785, 386.1783, 343.1358, 177.0324, 130.0494**
		7.7	618.2478 (-0.2)	**618.2462, 543.2150, 489.2045, 472.1780, 386.1776, 343.1356, 179.0498, 130.0501**
+2O-2H+GSH	$C_{30}H_{39}N_3O_{10}S$	6.2	634.2418 (-1.7)	**616.2314, 531.2153, 505.1992, 488.1705, 402.171, 385.1458, 359.1315, 341.1175, 308.0896,** 181.0323, **177.0325, 162.0216, 130.0493**
		6.3*	634.2421 (-1.2)	**616.2314, 559.2091, 505.1989, 488.1712, 402.1715, 385.1467, 359.13, 306.0757,** 274.1029
		6.5*	634.2423 (-0.9)	**616.2324, 559.2082, 505.1976, 402.1724, 385.1468, 359.1295, 306.0752**, 288.0646, 273.0949, 245.0631, 205.0327, 181.0317, **177.0324, 130.0493**
		6.7	634.2423 (-1)	**559.2108, 505.1992, 488.1727, 469.1783, 402.1741, 385.1456, 359.1302, 341.1203, 327.1589**, 181.0318, **130.0492**
+O-4H+2GSH	$C_{40}H_{54}N_6O_{15}S_2$	5.1*	462.1624^{+2} (1.5)	**616.2256, 541.1960, 487.1875, 470.1597,369.1497, 384.1620, 369.1524, 341.1209, 308.0908,** 233.0580, **162.0214, 130.0495**
		5.5	462.162^{+2} (0.7)	**618.2459, 559.2083, 489.2025, 384.1590, 343.1319, 306.0799**, 233.0590, **130.0495**
		6	462.1622^{+2} (1.2)	**616.2289, 575.1839, 541.1964, 519.1606, 501.1466, 487.1881, 469.1777, 368.1686, 343.1336**, 199.0713, **177.0322**
+O-2H+NAC	$C_{25}H_{31}NO_6S$	8.55	474.194 (-1)	**432.1821, 369.1508, 343.1359, 311.1636**
		9.05	474.1947 (0.3)	**432.1829, 369.1512, 343.1364, 311.1639**, 293.1537, **162.0212**
		9.3	474.1945 (0)	**432.1829, 369.1516, 343.1362, 311.1640, 162.0214**
+2O-2H+NAC	$C_{25}H_{31}NO_7S$	8.3	490.1885 (-1.9)	**472.1805, 454.1690, 430.1670, 311.1646,** 291.1380, **162.0211**
+O-4H+2NAC	$C_{30}H_{38}N_2O_9S_2$	7.3	635.2085 (-1.1)	**474.1959, 430.1672, 369.1585,** 291.1372, **162.0213**
		7.9	635.2086 (-0.9)	**430.1672, 369.1587,** 291.137, **162.0213**
